# Integration of Computer Vision and Wireless Networks to Provide Indoor Positioning

**DOI:** 10.3390/s19245495

**Published:** 2019-12-12

**Authors:** Jaime Duque Domingo, Jaime Gómez-García-Bermejo, Eduardo Zalama, Carlos Cerrada, Enrique Valero

**Affiliations:** 1 ITAP-DISA, University of Valladolid, 47002 Valladolid, Spain; jaigom@eii.uva.es (J.G.-G.-B.); ezalama@eii.uva.es (E.Z.); 2 Departamento de Ingeniería de Software y Sistemas Informáticos, ETSI Informática, UNED, C/Juan del Rosal, 16, 28040 Madrid, Spain; ccerrada@issi.uned.es; 3 School of Engineering, The University of Edinburgh, King’s Buildings, Edinburgh EH9 3FB, UK; e.valero@ed.ac.uk

**Keywords:** indoor positioning, WPS, RGB cameras, WiFi, *fingerprint map*, trajectory, IPS, computer vision

## Abstract

This work presents an integrated *Indoor Positioning System* which makes use of WiFi signals and RGB cameras, such as surveillance cameras, to track and identify people navigating in complex indoor environments. Previous works have often been based on WiFi, but accuracy is limited. Other works use computer vision, but the problem of identifying concrete persons relies on such techniques as face recognition, which are not useful if there are many unknown people, or where the robustness decreases when individuals are seen from different points of view. The solution presented in this paper is based on an accurate combination of smartphones along with RGB cameras, such as those used in surveillance infrastructures. WiFi signals from smartphones allow the persons present in the environment to be identified uniquely, while the data coming from the cameras allow the precision of location to be improved. The system is nonintrusive, and biometric data about subjects is not required. In this paper, the proposed method is fully described and experiments performed to test the system are detailed along with the results obtained.

## 1. Introduction

*Indoor Positioning Systems* (IPS) are taking on a key role in many domains, creating multiple opportunities in the era of artificial intelligence. Tracking the movement of robots/people and the use of data engineering makes it possible to optimize energy and costs and improve user experience by means of a proactive and reactive environment. These systems are also useful in improving medical applications.

Among active localization techniques, *WiFi-based Positioning Systems* (WPS) are a well-known option for tracking the movements of people in indoor environments. WPS is a geolocation system that uses the characteristics of nearby WiFi hotspots and other wireless access points to discover where a device like a smartphone is located. Recent WPSs have obtained an average error of 1.67 m [1,2], which is not very accurate for people tracking. Due to the error produced by these techniques, other systems have been developed. Technologies based on computer vision offer a different approach to the localization problem, but are not able to identify people easily. In these systems, identification is carried out by means of face detection [3] and recognition [4], appearance features [5], or color or patterns code [6]. Computer vision techniques have problems with occlusions and they need information about the users in the system.

A previous work of the authors [7] presented an indoor positioning system to estimate the location of people navigating complex indoor environments. The developed technique combines WiFi and RGB-D cameras in complex inhabited environments, consisting of various connected rooms, where people are moving freely. However, that paper considered that the number of people detected by WiFi positioning and detected by RGB-D needed to be equal, and required the installation of RGB-D cameras, such as Kinect v2—not usually available in common environments.

A new approach is presented in the present paper that combines WiFi and RGB cameras instead of RGB-D sensors, which results in a much simpler infrastructure requirement. A proper transformation to get a 3D model, based on the camera calibration parameters, is also used. The proposed approach is based on the method presented in [8] to extract the bird’s-eye view of the scenario and people’s trajectories. However, the method presented in [8] is restricted to the localization and tracking of people in single rooms and does not identify them. The method has been extended for use in more complex environments, such as hospitals or office environments, where there may be several rooms. To identify people and improve the localization, a new algorithm that integrates visual data with WiFi positioning is proposed.

The article is structured as follows: Section 2 explores the state-of-art of the technologies considered in this paper. Section 3 shows how the system works with RGB, WiFi, and the process of integrating both technologies. In Section 4, the different experiments and results carried out on people navigating indoors are reported and the results obtained are discussed. Finally, Section 5 notes the advantages and limitations of the presented system and suggests future developments based on this method.

## 2. Overview of Related Work

Modern IPSs use different technologies to track the location of persons. For these technologies, Koyuncu and Yang [9] suggest four different groups: (1) IPSs based on infrared or ultrasonic distances; (2) based on signal triangulation or Bluetooth/WiFi/RFID *fingerprint map*; (3) based on computer vision or the combination of such technologies as RFID or WiFi; and (4) inertial technologies (e.g., accelerometers and gyroscopes) or other types of sensor.

A survey of an optical indoor positioning system is presented in [10], where the authors classify the existing alternatives, according to the reference used, to figure out the position of users in the scene (e.g., 3D models, images, coded markers, or projected patterns). Different works have been developed in computer vision to track people positioning. Some of these, such as OpenPTrack [11] or the systems presented by Saputra et al. [12], Sevrin et al. [13], or Nakano et al. [14], consider a multicamera solution based on the use of RGB-D sensors. Others, like the works published by Mohedano et al. [8] or Elhayek et al. [15], follow an approach using common RGB cameras. Vision-based solutions can track people, but do not easily identify concrete persons. Other techniques, such as face recognition [4] or pattern recognition [6], look for a solution to the problem of identification.

*WiFi Positioning Systems* are mostly founded on the *fingerprinting* technique [16,17]. The *Received Signal Strength Indication* (RSSI) is used to generate a map of the environment with 2D coordinates and the values of signals received by different *Access Points* (APs), such as routers. A recent comparison between these systems has been presented by He and Chan [18]. Among the most advanced techniques, the authors explain how to make use of temporal or spatial signal patterns, user collaboration, and motion sensors.

Torres-Sospedra et al. [19] have recently presented the comparison between results obtained in IPIN (2014–2017) and Microsoft ISPN (2014–2017). The best results are obtained with a LIDAR system (IPIN 2017), which has an error below 0.1 m. This system is not available in small devices and has a bigger cost than other solutions. A WiFi fingerprinting-based system using a Bayesian filter was the winner of the infrastructure-free category in MS-ISPN 2014, obtaining an average error of 1.67 m [1,2]. Infrastructure-free solutions do not require modifications in the scenario. In IPIN 2016, Guo et al. [20] presented a WiFi and a *Pedestrian Dead Reckoning*-based system (PDR), which considers factors such as speed to calculate the next location starting from a fixed known position. An error of 1.5 m was obtained. Other authors [21,22,23] have also studied this combination of technologies, WiFi and PDR, but considering several limiting problems, such as the variation of WiFi signals and the drift of PDR. Formulating the sensor fusion problem in a linear perspective and using a Kalman filter, an average localization accuracy of 1 m was obtained. It is important to note that WiFi systems using the 2.4 GHz range obtained better results than others [1].

The combination of different technologies is a way of improving the efficiency of traditional WPSs [18,24,25]. The integration of data from RGB-D sensors and WiFi signals to provide more accurate positioning was proposed in [7]. However, the installation of RGB-D sensors is not usual in buildings and, in addition, it is not economical. The patent presented by Barton et al. [26] cited this previous work, integrating RGB cameras and WiFi signals by posing a theoretical idea of computing a proximity parameter to associate a person detected by a camera and the location data of a wireless device. This approach is prepared for identifying people using smartphones by means of cameras, but it does not cover a whole linear problem of multiple users, considering the trajectory of people in WiFi and RGB. Other hybrid systems combine WPS with such technologies as Bluetooth [27], RFID [28] or GSM [29], or Bluetooth beacon-based crowdsourced landmarks with inertial sensors [30] or GPS with inertial sensors [31]. A different approach is followed by Biswas and Veloso [32], who use a different technology (laser range-finder, RGB-D, or RSSI signals) depending on the position of a robot.

The IPSs are a useful way of creating reactive and proactive environments to enrich the experience of users. Several authors, such as Tsetsos et al. [33], Dudas et al. [34], Matuszka et al. [35], or Lee et al. [36] have integrated indoor navigation with semantic web technologies that focus on user activities.

The work presented in this article considers the trajectory of people in WiFi and RGB as a linear problem and calculates an association matrix to improve WiFi location with computer vision. RGB stereo-vision algorithms are used to track people in 3D and a novel algorithm integrates different numbers of people detected by RGB/WiFi.

## 3. Analysis of the System

This section is composed of three subsections: Section 3.1 explains how the system works to extract people’s trajectories from the RGB cameras. Section 3.2 presents how WiFi positioning works to obtain their respective trajectories. Section 3.3 illustrates the proposed integration between these two technologies: WiFi and RGB.

### 3.1. People-Tracking in RGB

Instead of developing a different approach to human detection in 2D using RGB, the problem is solved by considering a well-known alternative based on neural networks. Among the previously developed systems, some rely on the use of *Haar filters* [37] or *Histograms of Oriented Gradients* [38]. Recent systems use *Deep Convolution Neural Networks* (DCNN), such as the *Faster RCNN Inception V2 COCO Model* [39]. All of them are available in OpenCV, working simultaneously with TensorFlow. Systems based on DCNN offer better results, detecting more people with a lower number of false positives. However, these systems are slower, requiring more time to process images. Considering that, the experiments have been carried out with a 2-s time stamp, so DCNN systems are feasible for providing useful data. Concretely, *Faster RCNN Inception V2* has been used to extract people from images. Results obtained in this stage are a set of rectangles framing the different people detected.

**Figure 1:**
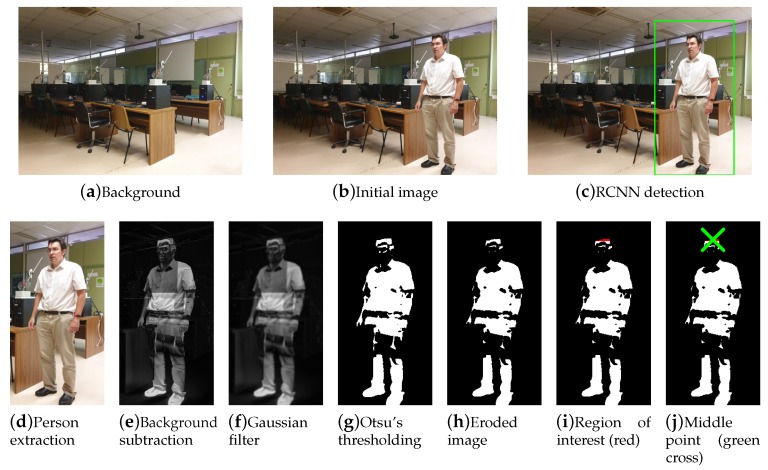
Process of obtaining the position of the person in 2D.

**Figure 2:**
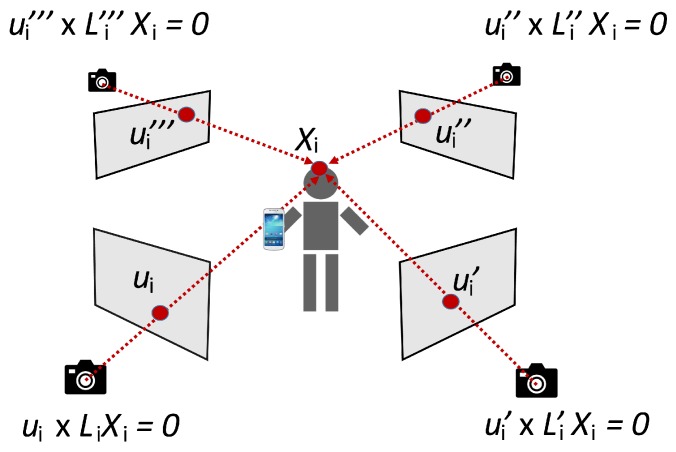
Components of the Linear Triangulation Method.

For each rectangle obtained in the previous stage, a region has been extracted by comparing the image with the static background of the scenario (obtained by each camera before the experiments). Otsu-based segmentation on the Gaussian-filtered images, followed by some morphology, produces a final binary image of each person, as shown in Figure 1. The top region of points is then used to obtain the coordinates of the person, provided that the region has more than a particular number of points. These coordinates, corresponding to the top of the person’s head, represent a better approach to avoid variances due to static foreground occlusions produced at other points [8]. 3D coordinates are subsequently obtained by means of a Linear Triangulation Method [40] applied to the 2D coordinates obtained from each camera. This method is carried out in 2 steps:

1. A first step consists of determining the camera projection matrix, *L*, associated to each camera, before the experiments. It is important to mention that all cameras involved in the process must satisfy the epipolar constraint. The *L* matrix allows the 3D coordinates to be related to the 2D image perceived by the camera, according to $LX_{i}=u_{i}$, where $X_{i}$ are the 3D coordinates of a given point and $u_{i}$ are its corresponding image coordinates. To obtain *L*, six different normalized points are registered in the scenario, indicating the 3D coordinates with respect to the room and 2D position in each image obtained by its respective camera. A *Direct Linear Transformation* (DLT) algorithm estimates *L* from 2D/3D coordinates.
2. A second step, performed during execution time, allows 3D $X_{i}$ coordinates to be identified from a set of different 2D points for each camera: $u_{i}$, $u_{i}^{{}^{\prime }}$, $u_{i}^{{}^{\prime \prime }}$, etc., (see Figure 2). Each camera has its respective *L* matrix, relating 3D common coordinates with their respective 2D ones, such as $LX_{i}=u_{i}$ or $L^{{}^{\prime }}X_{i}=u_{i}^{{}^{\prime }}$. These expressions are equivalent to $u_{i}$ x $LX_{i}=0$ or $u_{i}^{{}^{\prime }}$ x $L^{{}^{\prime }}X_{i}=0$. Based on these expressions, each perspective camera model produces two equations. The complete set of equations represents an overdetermined homogeneous system of linear equations which is solved with SVD, obtaining a 3D point that is optimal in a least squares sense.
  When cameras detect more than one person in a room, the best geometrical association between persons detected by different cameras is the combination that minimizes the residual value *r* of the previous overdetermined linear equation [41,42].

**Figure 3:**
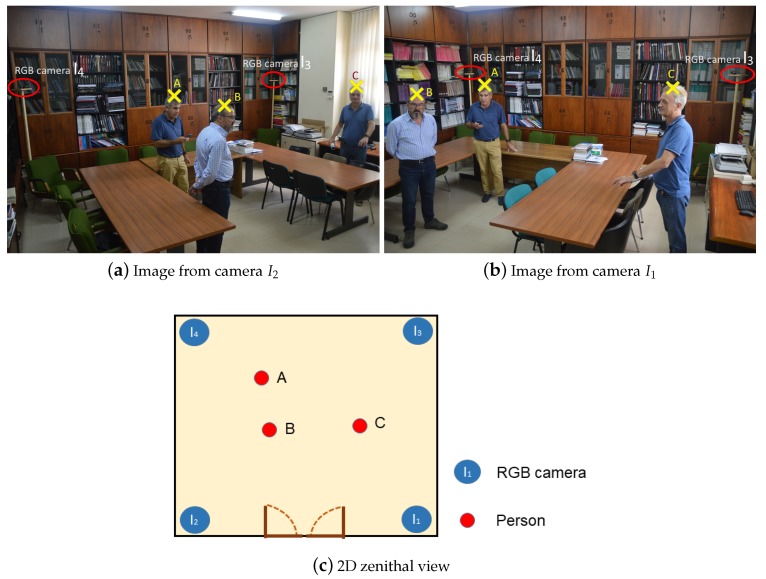
Process of obtaining the position in 2D zenithal view. $I_{3}$ and $I_{4}$ are in red circles.

Once the 3D coordinates have been obtained, they are transformed into a 2D UCS *Universal Coordinates System*. This 2D map is the aerial view of people obtained from different cameras. Figure 3 shows two images obtained by cameras $I_{1}$ and $I_{2}$ and its representation in the 2D zenithal view.

Finally, the trajectory between consecutive time stamps is computed by comparing results obtained in the 2D aerial view. As soon as new coordinates are calculated, they are compared with the previous ones to track persons. People follow a path where two points are part of the same trajectory of the person if both are situated at less than a concrete distance.

### 3.2. People-Tracking in WiFi

A *fingerprint map* is a table that relates a set of signal intensities (RSSI) with the 2D aerial coordinates where they were obtained. Once a *fingerprint map* has been created, it is possible to estimate the position of a new set of RSSI values calculating the Euclidean distance with the previous groups. The table includes the *Basic Service Set Identifier* (BSSID) of the Access Point (AP, e.g., router), the corresponding *Received signal strength indication* (RSSI), and the 2D coordinates *x* and *y*.

There are different ways of creating the *fingerprint map* [43,44,45], but a convenient method is using RGB-D cameras to register the 2D coordinates. They are more accurate than other solutions, such as normal cameras or manual measurements. For this reason, the creation of the *map* has been carried out using RGB-D sensors. Once the *map* has been created, the positioning process only relies on the use of normal RGB cameras.

**Figure 4:**
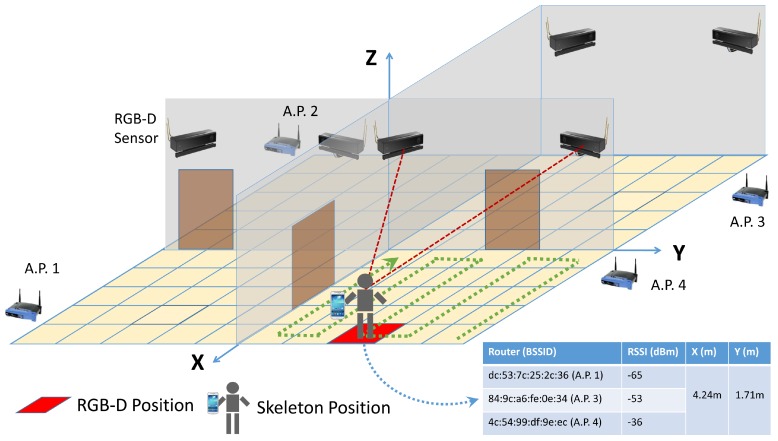
Creation of the WiFi *fingerprint map*.

During the creation of the *map*, a user freely moves around the environment. Figure 4 shows a person who moves following a given trajectory (in green) and the created table. The RSSI values from the cell phones, received from the APs, together with their position estimated by the RGB-D sensors, are recorded in the *map* every time the user clicks a button of an application developed for this purpose. RSSI values from 16 different APs have been recorded. Considering that the Kinect v2 returns the coordinates of different body joints, the coordinates of the neck have been considered, as they are the joint less prone to be occluded by elements in the scenario. In addition, a general universal coordinate system has been created for the full scenario.

As the map is created exclusively by one person, two RGB-D sensors return the coordinates of the same person. When the data collection ends, in order to simplify the positioning process without significant loss of precision, another essential task is carried out at the end of this stage: the complete scenario is divided into cells (e.g., 1 × 1 m) and RSSI data are grouped in each cell using the corresponding position of the 2D aerial coordinates. An RSSI vector is created for each cell, pairing each component to the centroid for all of the RSSI measurements of a certain AP. RSSI scans are grouped according to the distance between their original associated 2D coordinates and the coordinates of the centre of each cell. This step reduces the size of the *fingerprint map* to improve the performance of the system and obtain more reliable RSSI values, avoiding variations in the measurements.

It is worth noting that RGB-D sensors are not strictly necessary to create a *fingerprint map*. They have been used for convenience in our experiments, but will no longer be required during the system operation.

People positioning is carried out by comparing the RSSI values obtained from the smartphones with the RSSI centroids of the map and returning the coordinates of the virtual cell with less difference. These comparisons are implemented by the Euclidean distance between the RSSI vectors [46]. The trajectory of each person with WiFi is related to their smartphone.

However, systems based on WiFi do not deliver accurate results in indoor environments. The integration with a different technology, such as computer vision, improves the accuracy and offers promising results.

### 3.3. RGB and WiFi Integration

**Figure 5:**
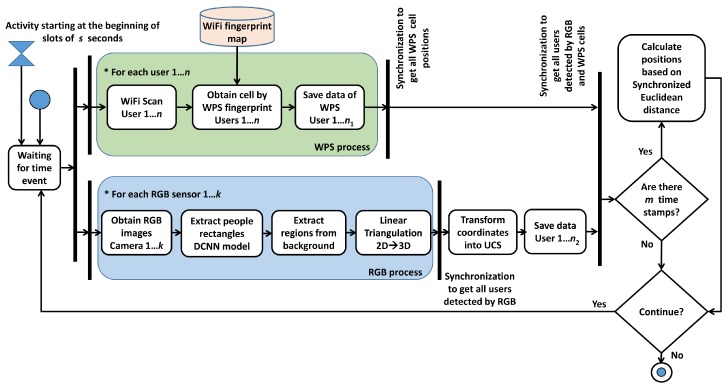
Diagram of the system.

WiFi positioning is improved with computer vision by associating the trajectory of a person obtained by WiFi and the trajectory of the same person by RGB. Initially, there is a set of RGB and WiFi trajectories that are disassociated, that is, the system does not know which WiFi trajectory an RGB one corresponds to, and vice versa. The problem presented in this section solves the association regardless of whether there is a different number of people detected by each of the technologies. The diagram of the proposed system is shown in Figure 5, describing the steps explained in the previous sections for RGB and WPS. The association between both trajectories, represented in the schema as *Calculate Positions based on Synchronized Euclidean distance*, is explained below.

The integration is modeled as a linear problem, where the goal is to optimize an objective function, $\min D^{T}\cdot S$, subject to some linear inequality constraints. *D* represents the Euclidean distances between each pair of RGB and WiFi trajectories, while *S* represents the binary *Matching Matrix*, which associates the WiFi trajectories of all people with their respective RGB ones. There are different restrictions to contemplate the case where it is possible to detect a different number of people using each technology. The variables used and details on the objective function and the different restrictions are explained next:

- Let $n_{1}$ be the number of persons detected by WiFi and $n_{2}$ be the number of persons detected by RGB.
- Let *m* be the number of time stamps to be considered and *T* the time stamp duration. Both positioning systems provide their corresponding position measurement for a concrete number of people in each time stamp: $n_{1}$ people in WiFi and $n_{2}$ in RGB.
- Let $P_{i}\left(kT\right)$ be the position of the person *i* provided by the WiFi at the time stamp *kT*, and $P_{j}^{{}^{\prime }}\left(kT\right)$ be the position of the person *j* provided by the RGB cameras at the time stamp *t*. The problem consists in determining, for all the time stamps considered, the best trajectory estimation of people by determining the data pairing set (i,j) that corresponds to individuals detected by WiFi (smartphone signal) and RGB cameras.
- Let $s_{ij}$ be the binary element of the *Matching Matrix, S*, in row *i*, column *j*. This matrix links the WiFi trajectories with their respective RGB, being $s_{ij}=1$ if the person is detected by the WiFi and cameras, and 0 otherwise.

The problem is approached as an optimal combination of WiFi and RGB trajectories for all people detected in a set of time stamps. The solution to the algorithm is to find the matrix *S*, valid for the complete dataset, such that the best combination is achieved when the sum of the distances from all possible trajectories is minimum, as shown in

(1) $$\begin{array}{c} \min\sum_{i=1}^{n_{1}}\sum_{j=1}^{n_{2}}s_{ij}\cdot \sum_{k=1}^{m}d_{E}(P_{i}\left(kT\right),P_{j}^{{}^{\prime }}\left(kT\right)), \end{array}$$

where $d_{E}$ represents the Euclidean distance between the coordinates of a person detected in RGB and WiFi. For WiFi, the coordinates used are the centroids of the virtual cells in the 2D aerial map. This equation minimizes the *Synchronized Euclidean distance* [47], computing the sum of the distances between each pair of points of WiFi and RGB.

The problem is subject to the following restrictions:

(2) $$\begin{array}{c} \;\;\;\;\;\;\;\mathrm{if}\;n_{1}>n_{2},\left\{\begin{array}{c} \forall i=1,\ldots ,n_{1}:\sum_{y=1}^{n_{2}}s_{iy}\leq 1\\ \forall j=1,\ldots ,n_{2}:\sum_{x=1}^{n_{1}}s_{xj}=1\\ \sum_{x=1}^{n_{1}}\sum_{y=1}^{n_{2}}s_{xy}=n_{2},\;\;\;\;\;\;\;\;\;\;\;\;\;\;\;\;\; \end{array}\right. \end{array}$$

(3) $$\begin{array}{c} \;\;\;\;\;\;\;\mathrm{if}\;n_{1}=n_{2},\left\{\begin{array}{c} \forall i=1,\ldots ,n_{1}:\sum_{y=1}^{n_{2}}s_{iy}=1\\ \forall j=1,\ldots ,n_{2}:\sum_{x=1}^{n_{1}}s_{xj}=1\\ \sum_{x=1}^{n_{1}}\sum_{y=1}^{n_{2}}s_{xy}=n_{1},\;\;\;\;\;\;\;\;\;\;\;\;\;\;\;\;\; \end{array}\right. \end{array}$$

(4) $$\begin{array}{c} \;\;\;\;\;\;\;\mathrm{if}\;n_{2}>n_{1},\left\{\begin{array}{c} \forall i=1,\ldots ,n_{1}:\sum_{y=1}^{n_{2}}s_{iy}=1\\ \forall j=1,\ldots ,n_{2}:\sum_{x=1}^{n_{1}}s_{xj}\leq 1\\ \sum_{x=1}^{n_{1}}\sum_{y=1}^{n_{2}}s_{xy}=n_{1}.\;\;\;\;\;\;\;\;\;\;\;\;\;\;\;\;\; \end{array}\right. \end{array}$$

In the case of Equation (2), not all people detected by WiFi are detected by cameras. In the case of Equation (3), all people detected by WiFi are detected by cameras. In the case of Equation (4), there are more people detected by cameras than from WiFi.

The problem is solved using a Mixed Integer Linear Programming (MILP) solver, based on the revised simplex method and the Branch-and-bound method for the integers [48]. The Branch-and-bound algorithm finds a value *x* that minimizes a function f(x) among some set *C* of candidate solutions. It recursively divides the search space into smaller spaces and minimizes f(x) on these spaces. Instead of performing a brute-force search, the Branch-and-bound algorithm keeps in memory the bounds on the minimum to be found, and uses these bounds to prune the space, eliminating candidates that will not be optimal.

**Figure 6:**
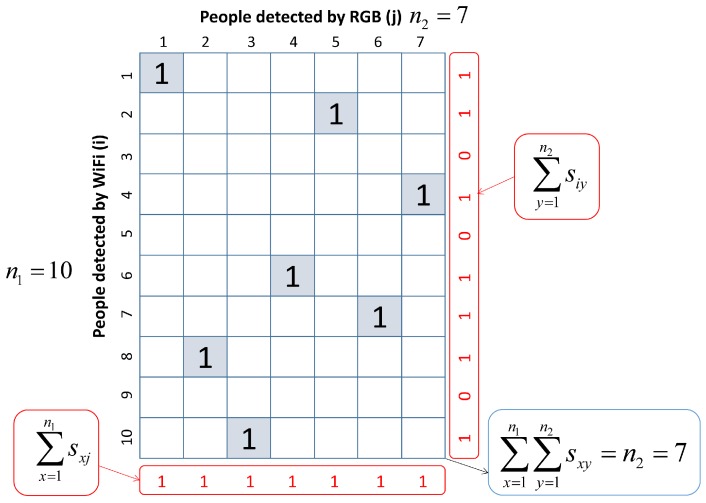
Matrix *S*, matching each WiFi/RGB trajectory.

The result of the algorithm produces the matrix *S*, matching each WiFi trajectory with the corresponding RGB one. If there is no RGB trajectory for a person, the system uses the WiFi trajectory as it is the one directly obtained by the smartphone. Figure 6 shows an example of matrix *S*, matching a different number of people detected by WiFi, $n_{1}=10$, and RGB, $n_{2}=7$.

Continuous tracking in RGB is carried out using the 2D zenithal coordinates of people. When new coordinates are calculated, they are compared with the previous ones to find those with the smallest Euclidean distance. This principle is used when a person leaves a room and enters a different one. When a person is occluded or overlapped by another person for a given camera, the position is calculated from the remaining cameras given that DLT/SVD algorithms can work with only two cameras. Continuous tracking in WiFi is directly obtained by smartphones because each set of RSSI scans is associated to a concrete device. After the algorithm, each RGB trajectory is associated to a WiFi one in *m* time stamps. For this association, data from $t_{k}$ to $t_{k+m-1}$ time stamps are considered. In the next time stamp, the algorithm moves one slot of time and considers data from $t_{k+1}$ to $t_{k+m}$.

## 4. Experiments and Discussion

**Figure 7:**
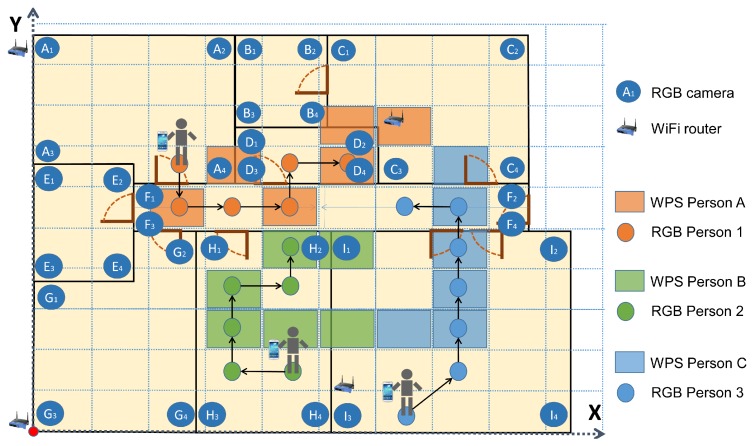
Scenario of the system.

The scenario is an 80 m${}^{2}$ office, with 8 rooms and a central corridor, as shown in Figure 7. Kinect v2 cameras were used to generate the WiFi *fingerprint map*. Afterwards, they were replaced by conventional RGB cameras, as only RGB images have been considered for computer vision positioning.

**Figure 8:**
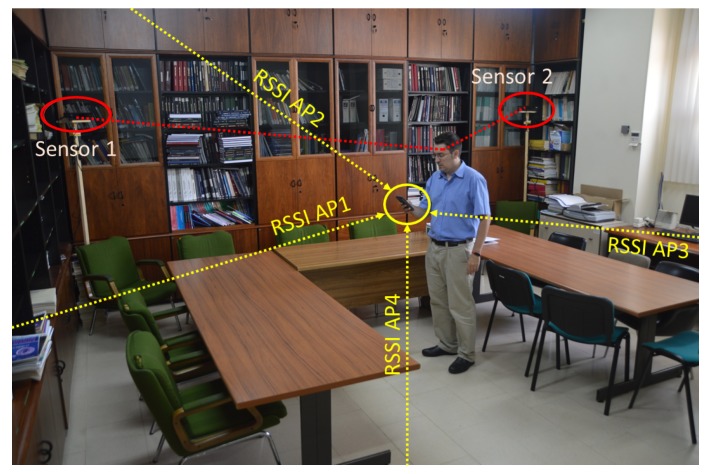
Creating the WiFi *fingerprint map*.

To create the *fingerprint map*, RSSI values were obtained from a person at the same time as skeletons were received by the Kinect sensors. Whenever the person clicked a button, a complete scan of APs and skeletons was done. Then, head coordinates and RSSI values were stored in a database. Figure 8 shows one person creating the *fingerprint map* with 2 RGB-D sensors, moving them between different rooms.

A set of 36 RGB cameras of different models were deployed in the scenario. The calibration of the RGB cameras, matrix *L*, was calculated using eight different normalized points registered in each room, considering the 3D coordinates with respect to the room and the 2D position in each image obtained by its respective camera. The points were visible from the different cameras of the room. Once the group of cameras had been calibrated, the error in obtaining the coordinates of a concrete fixed point in the scenario was 0.03 m. The error in locating a person was 0.13 m, similar to other works [15].

**Figure 9:**
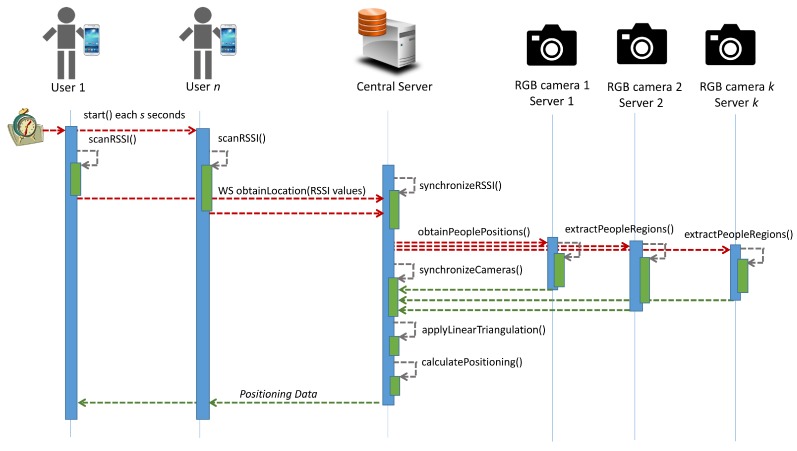
Sequence diagram of the system.

As shown in Figure 9, an Android-based application was created. In this application, different users called a web service named *obtainLocation()*, passing the RSSI values at a synchronized time. Once all RSSI values had been received, the central web server called different services to obtain 2D coordinates of people (*obtainPeoplePositions()*).

For performance reasons, each camera was connected to a dedicated server to compute the *Faster RCNN Inception V2 model* [39] (*extractPeopleRegions()*). Every 2 seconds, the application sent RSSI data to a central web server, which was connected to each camera by means of the intermediate computer. A 2-second slot between time stamps was valid for the experiments. Finally, after the synchronization of the results, Linear Triangulation (*applyLinearTriangulation()*) and the positioning algorithm (*calculatePositioning()*) were executed.

**Figure 10:**
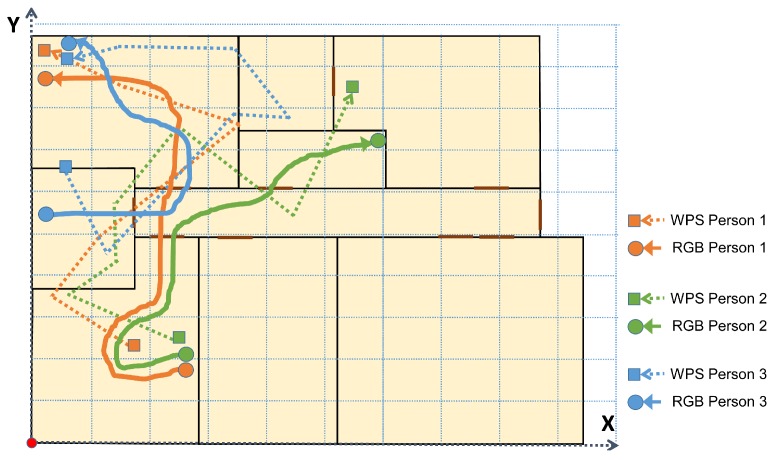
Real trajectories obtained during experiment.

Four people participated directly in this experiment, following previously established trajectories. A value of m=10 time stamps was used for the experiments. A bigger value improves the efficiency of the algorithm, but requires excessively long trajectories. At the same time, a high value of *m* increases computing cost. m=10 has been found to produce good results with a short trajectory. The system properly associated the RGB and WiFi trajectory in 99.10% of cases. When a person was not detected by the RGB cameras, the success was 96.80% for associating the rest of the trajectories. Different cases were evaluated for two, three, and four people. Figure 10 shows three people following a concrete trajectory. Two of them (persons 1 and 2) go together until a point where they separate.

Table 1 presents data related to person 1 in the previous trajectory, where the RSSI values obtained by smartphone and the RSSI values corresponding to the WPS cell of the *fingerprint map* are shown. RGB coordinates in the UCS are also presented.

These results can be extrapolated for a greater number of people. To this end, after the completion of the real experiment, 50 different WiFi and RGB trajectories were obtained. Each person moved around the scenario following different trajectories that were recorded in a database with their WiFi and RGB information. When data collection finished, these 50 trajectories were associated, indicating the WiFi trajectory corresponding to the RGB one. The efficiency of the system was validated, randomly combining these 50 trajectories from 5 to 20 people, producing more than 1 million different combinations. The experiments considered the trajectory of different numbers of people, taking into account both technologies during 10 time stamps of 2 s. In every test, a random WiFi trajectory was selected for each person. RGB and WiFi trajectories were disassociated and the evaluation checked if the WiFi trajectory was again associated with the corresponding RGB one. Table 2 shows the success of associating all the trajectories of the people detected by WiFi with their respective trajectory in RGB. Considering that the 50 trajectories include their respective WiFi/RGB data, the evaluation of the system is performed by determining whether the WiFi trajectory associated to the RGB is the same as the one recorded for that trajectory. When a lower number of people is detected by a concrete technology, this reflects that all users of that technology have been properly linked to it. When the number of people detected by both technologies is equal, the success rate increases because there are fewer false-positives. People not linked properly with RGB obtain the location based on WiFi.

A Mixed Integer Linear Programming (MILP) solver, based on the revised simplex method and the Branch-and-bound method for the integers [48], has been used to solve the problem. The algorithm was run on a server with an Intel i9-9900K processor and 32 GB of RAM. It took from 0.6 ms to associate the simplest situations ($n_{1}<10$ and $n_{2}<10$) to 1.95 ms for the most complex one ($n_{1}=20$ and $n_{2}=20$).

The computational cost follows an exponential function $t=0.08\cdot 1.16^{n}+0.42$, where *t* represents the processing time in milliseconds and *n* the maximum number of users. When n=50 people, the processing time is 134 ms, which works properly in aspects of time. However, in an 80 m${}^{2}$ scenario, that amount of people is overcrowded. For larger scenarios with more than 50 people, the scenario must be divided using the APs detected by smartphones. A person will be associated in the zone corresponding to the APs detected.

**Figure 11:**
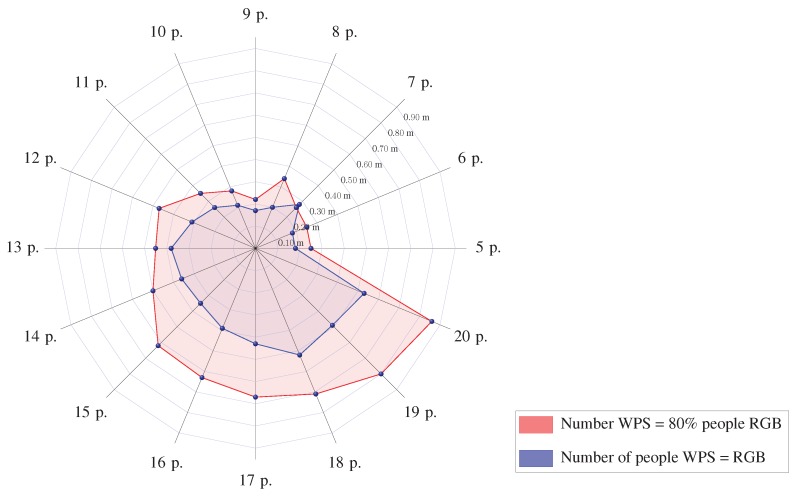
Average error of the system (in meters) for different numbers of people.

Considering a verified average distance error of 0.13 m for RGB and 2.0 m for WiFi, an average error of 0.53 m has been estimated for 20 people. This error is calculated by multiplying the positive associations by the RGB average error and the rest by the WiFi error. In Figure 11, in blue, an average error in meters is shown, considering the same number of people being detected simultaneously by WiFi and RGB. In red is the error computed assuming that WPS only works for 80% of the people detected by the RGB cameras, which could correspond to people not carrying their smartphones. In this case, the number of people detected by RGB corresponds to the radius.

There are positioning systems that make use of computer vision to track people, such as [6], where the authors present a system to follow a group of workers using the color of their hardhats, or OpenPTrack [11], a scalable and multicamera solution. However, these systems do not identify concrete persons. Computer vision produces accurate results, but it requires other techniques, such as face recognition [4] or appearance features [5], to solve the problem of identification. The comparison between the results obtained and the existing ones has to be made with other systems that are able to identify people, such as WiFi fingerprinting-based systems. Concretely, the error obtained (0.53 m) is lower than previous exclusive WiFi fingerprinting-based systems, such as [2] (1.67m), [20] (1.5 m), or [23] (1 m). It should be noted that the presented algorithm can be easily integrated with other computer vision systems, such as OpenPTrack [11].

## 5. Conclusions

This work presents an integrated indoor positioning system for use in complex environments where multiple people carry smartphones and which makes use of computer vision to provide an accurate position. The system uses WiFi positioning and several RGB cameras installed in each room to reduce occlusions. The algorithm is able to manage a different number of people detected by RGB/WiFi and is focused on tracking people, but it could easily be extended to the localization of nonhuman targets such as mobile robots, *Automated Guided Vehicles* (AGVs), etc. on work floors.

The system successfully links the trajectory of all the people detected by the computer vision with their respective trajectory in 79% of cases for 20 persons moving freely around the scenario. Considering an average error for RGB/WiFi detection, a positioning error of 0.53 m has been calculated. Of course, the successes would decrease consistently if there was a large difference between the number of people detected by each technology. The error is, however, lower than previous exclusive WiFi fingerprinting-based systems, such as [2] (1.67 m), [20] (1.5 m), or [23] (1 m). The algorithm presented could be improved and integrated with these previous WPS methods or others which make use of inertial sensors or Kalman filters. It could also be integrated with algorithms based on Bluetooth, such as [30], which use Bluetooth beacon-based landmarks and exploits inertial sensors composed of an accelerometer, magnetometer, and gyroscope.

The experiments were carried out using common RGB cameras to obtain images and exploit the process of people-tracking by DCNN. Four cameras were installed in each room, covering an 80 m${}^{2}$ office. Although four cameras were used to improve the results, only two cameras are needed, since DLT/SVD algorithms can work with just two cameras. The difference in accuracy using two cameras with respect to four is ±0.02m, although they do not work so well with occlusions or overlaps and they may not capture all the angles of the room. Moreover, two Kinect v2 sensors were used only to create the WiFi *fingerprint map*, as this is a fast and accurate way to build it. It is important to note that, regarding scaling, the system is prepared to work in large environments. A computational cost has been analyzed, indicating a recommended limit of about 50 people per zone. In a large environment, when there are more than 50 people, the scenario would be split into different zones.

From the point of view of cost, the solution presented is an economical way to increase the performance of WPS in interiors. Previously installed surveillance cameras are candidates for implementing the RGB tracking while smartphones are widespread. There are multiple applications of the system, mainly focused on the creation of reactive and proactive environments to enrich the user experience. For example, the work [49] integrates an indoor positioning system with Europeana [50], a data source about artworks, to proactively show information about the pieces situated near a person inside a museum. Future works will consider the use of this system in more complex scenarios, such as medical or industrial environments or private facilities, deploying a complete set of cameras. 

## Figures and Tables

**Table 1:** Extraction of data for person 1 during experiments.

Time Stamp	RSSI Values (dBm)	RSSI *Fingerprint map* of Corresponding WPS Cell (dBm)	WPS Cell	RGB UCS Coordinates (m)
1	AP1:72.0; AP2:25.0; AP3:67.0; AP4:72.0; AP5:72.0; AP6:77.0; AP7:54.0; AP8:87.0; AP9:82.0; AP10:93.0; AP11:85.0; AP12:86.0; AP13:86.0; AP14:87.0; AP15:92.0; AP16:93.0;	AP1:66.96; AP2:36.52; AP3:67.11; AP4:65.74; AP5:69.92; AP6:79.96; AP7:57.14; AP8:84.77; AP9:83.46; AP11:87.12; AP12:89.39; AP13:89.9; AP15:92.14;	(2, 7)	(2.73, 7.01)
2	AP1:75.0; AP2:26.0; AP3:65.0; AP4:75.0; AP5:73.0; AP6:76.0; AP7:56.0; AP8:87.0; AP9:80.0; AP10:93.0; AP11:85.0; AP12:93.0; AP13:93.0; AP14:90.0; AP15:93.0; AP16:93.0;	AP1:66.96; AP2:36.52; AP3:67.11; AP4:65.74; AP5:69.92; AP6:79.96; AP7:57.14; AP8:84.77; AP9:83.46; AP11:87.12; AP12:89.39; AP13:89.9; AP15:92.14;	(2, 7)	(1.94, 8.05)
3	AP1:79.0; AP2:32.0; AP3:68.0; AP4:76.0; AP5:75.0; AP6:78.0; AP7:63.0; AP8:84.0; AP9:89.0; AP11:90.0; AP12:87.0; AP13:87.0; AP14:92.0; AP15:93.0; AP16:94.0;	AP1:66.96; AP2:36.52; AP3:67.11; AP4:65.74; AP5:69.92; AP6:79.96; AP7:57.14; AP8:84.77; AP9:83.46; AP11:87.12; AP12:89.39; AP13:89.9; AP15:92.14;	(2, 7)	(2.04, 7.21)
4	AP1:77.0; AP2:37.0; AP3:70.0; AP4:77.0; AP5:60.0; AP6:80.0; AP7:59.0; AP8:89.0; AP9:79.0; AP11:90.0; AP12:91.0; AP13:92.0; AP15:93.0; AP16:94.0;	AP1:66.96; AP2:36.52; AP3:67.11; AP4:65.74; AP5:69.92; AP6:79.96; AP7:57.14; AP8:84.77; AP9:83.46; AP11:87.12; AP12:89.39; AP13:89.9; AP15:92.14;	(2, 7)	(2.06, 7.19)
5	AP1:59.0; AP2:31.0; AP3:69.0; AP4:58.0; AP5:63.0; AP6:80.0; AP7:57.0; AP8:84.0; AP9:90.0; AP11:90.0; AP12:89.0; AP13:89.0;	AP1:66.45; AP2:33.32; AP3:68.55; AP4:66.59; AP5:67.95; AP6:77.86; AP7:56.45; AP8:85.95; AP9:83.27; AP11:86.0; AP12:87.65; AP13:86.82;	(1, 7)	(2.34, 6.54)
6	AP1:67.0; AP2:54.0; AP3:62.0; AP5:62.0; AP4:67.0; AP8:81.0; AP6:73.0; AP7:70.0; AP9:86.0; AP10:88.0;	AP1:63.81; AP2:42.18; AP3:61.81; AP5:67.63; AP4:64.09; AP8:82.8; AP6:79.36; AP7:58.09; AP10:91.0; AP9:84.8;	(2, 6)	(3.16, 3.61)
7	AP1:53.0; AP3:38.0; AP4:53.0; AP5:65.0; AP2:49.0; AP6:60.0; AP8:73.0; 8c:0c:a3:45:38:5f:82.0; AP7:65.0; AP10:84.0;	AP1:43.0; AP3:54.0; AP4:43.0; AP5:60.0; AP2:60.5; AP6:71.0; AP8:70.0; AP7:62.5; AP10:77.5;	(4, 3)	(1.50, 1.58)
8	AP1:51.0; AP3:42.0; AP4:51.0; AP5:62.0; AP2:52.0; AP6:61.0; AP7:64.0; AP8:74.0; AP10:82.0;	AP1:50.33; AP3:41.5; AP4:50.66; AP5:65.33; AP2:55.66; AP6:60.16; AP8:73.83; AP7:73.67; AP10:81.33;	(1, 2)	(1.10, 1.20)
9	AP1:57.0; AP3:43.0; AP4:57.0; AP5:62.0; AP2:55.0; AP6:61.0; AP7:64.0; AP8:71.0; AP9:89.0; AP10:74.0;	AP1:57.85; AP3:42.57; AP4:55.38; AP5:62.57; AP2:55.81; AP6:62.66; AP8:78.0; AP7:70.43; AP10:82.45; AP9:92.67;	(1, 1)	(1.04, 1.17)
10	AP1:50.0; AP3:37.0; AP4:50.0; AP5:60.0; AP2:51.0; AP6:65.0; AP7:64.0; AP8:74.0; AP10:78.0;	AP1:57.85; AP3:42.57; AP4:55.38; AP5:62.57; AP2:55.81; AP6:62.66; AP8:78.0; AP7:70.43; AP10:82.45; AP9:92.67;	(1, 1)	(0.28, 1.49)

**Table 2:** Association success rate (%) depending on a different number of people detected by WiFi or RGB.

WiFiRGB	5	6	7	8	9	10	11	12	13	14	15	16	17	18	19	20
**5**	97	97	93	92	86	81	68	89	77	80	85	67	81	75	50	71
**6**	96	98	95	89	92	77	75	93	81	79	72	84	71	45	62	56
**7**	92	92	92	90	91	89	84	83	81	70	82	73	69	75	66	62
**8**	88	95	90	96	95	92	77	81	82	84	51	80	77	65	60	68
**9**	84	74	88	77	98	91	88	72	84	77	71	68	51	66	71	32
**10**	81	81	80	82	88	96	76	82	83	75	73	52	80	48	67	45
**11**	81	72	86	76	78	93	93	84	83	90	70	72	42	50	43	43
**12**	89	82	84	72	78	87	88	90	88	80	74	83	76	71	66	58
**13**	82	76	80	79	80	69	80	80	86	85	81	73	78	73	58	70
**14**	76	74	73	63	64	80	78	73	83	88	85	79	71	69	65	58
**15**	75	77	75	81	72	71	65	69	84	82	88	85	71	71	64	65
**16**	81	65	66	69	74	70	55	61	58	71	80	86	81	66	70	61
**17**	71	45	68	57	54	68	50	51	68	64	73	66	84	57	47	56
**18**	75	76	43	41	61	36	46	67	52	71	58	70	67	79	74	74
**19**	50	74	64	35	61	51	57	62	56	57	52	63	69	62	81	58
**20**	61	48	65	62	58	31	54	56	63	58	49	61	65	52	60	79
